# 609. Utilization of a Pain and Sedation Guideline in a Burn Intensive Care Unit

**DOI:** 10.1093/jbcr/irag033.420

**Published:** 2026-04-06

**Authors:** Katie Ronis, Lindsay DeSantis, Laurin Proctor, Nicholas Starr, Samantha Allbritton, Gregory Margolin, Duried Kassab, Lily Daniali, Ryan Endress, Wojciech Przylecki, Benson Pulikkottil

**Affiliations:** HCA HealthONE Swedish, Englewood, CO; HCA HealthONE Swedish, Englewood, CO; HCA HealthONE Swedish, Englewood, CO; HCA HealthONE Swedish, Englewood, CO; HCA HealthONE Swedish, Englewood, CO; HCA HealthONE Swedish, Englewood, CO; HCA HealthONE Swedish, Englewood, CO; HCA HealthONE Swedish, Englewood, CO; HCA HealthONE Swedish, Englewood, CO; HCA HealthONE Swedish, Englewood, CO; HCA HealthONE Swedish, Englewood, CO

## Abstract

**Introduction:**

Literature demonstrates sedation and analgesia protocol effectiveness in Intensive Care Unit (ICU) populations, with benefits including reductions in continuous infusion days, ventilator days, and overall ICU length of stay. Burn specific literature surrounding protocolization of sedation and pain management is sparse. This study aimed to evaluate the effectiveness of a standardized guideline for pain and sedation management in a Burn ICU.

**Methods:**

A retrospective observational study was conducted, assessing patients pre and post implementation of a pain and sedation guideline. Patients admitted within a 21-month period were evaluated for inclusion. The guideline outlines treatment for background pain, breakthrough pain, procedural pain, and sedation management for mechanically ventilated burn patients. Data collected included demographic and injury characteristics, pain and sedation medication use, delirium incidence, ventilator days, and ICU and hospital length of stay. Patients who expired during hospitalization were excluded from analysis.

**Results:**

A total of 21 patients were included (pre-guideline 13, post-guideline 8). Included patients had a mean burn size of 30.9% TBSA pre-guideline and 32.9% TBSA post-guideline (*p*=.68). No statistical difference was found in the outcomes studied. Delirium days were 4 days pre-guideline and 1.24 days post-guideline (*p*=.11). Duration of mechanical ventilation was 22.8 days pre-guideline and 19.1 days post-guideline (*p*=.64). ICU length of stay was 37.9 days pre-guideline and 37.1 days post-guideline (*p*=.9). Hospital length of stay was 63.6 days pre-guideline and 55 days post-guideline (*p*=.57).

**Conclusions:**

Implementation of a standardized sedation and pain management guideline in mechanically ventilated burn patients was not associated with statistically fewer days of delirium, days of mechanical ventilation, and length of stay. However, there was a noted trend towards a reduction in these outcomes.

**Applicability of Research to Practice:**

Due to the unique pharmacokinetics of burn patients, structured guidelines can help support critical care providers in the management of pain and sedation.

**Funding for the study:**

N/A.

